# Adaptive fractional order sliding mode control for Boost converter in the Battery/Supercapacitor HESS

**DOI:** 10.1371/journal.pone.0196501

**Published:** 2018-04-27

**Authors:** Jianlin Wang, Dan Xu, Huan Zhou, Tao Zhou

**Affiliations:** 1 School of mechanical engineering, Xi’an Jiaotong University, Xi’an, ShanXi, China; 2 Department of college of science, Ningxia medical university, Yinchuan, NingXia, China; Chongqing University, CHINA

## Abstract

In this paper, an adaptive fractional order sliding mode control (AFSMC) scheme is designed for the current tracking control of the Boost-type converter in a Battery/Supercapacitor hybrid energy storage system (HESS). In order to stabilize the current, the adaptation rules based on state-observer and Lyapunov function are being designed. A fractional order sliding surface function is defined based on the tracking current error and adaptive rules. Furthermore, through fractional order analysis, the stability of the fractional order control system is proven, and the value of the fractional order (λ) is being investigated. In addition, the effectiveness of the proposed AFSMC strategy is being verified by numerical simulations. The advantages of good transient response and robustness to uncertainty are being indicated by this design, when compared with a conventional integer order sliding mode control system.

## Introduction

Hybrid electric energy storage system (HESS) is an advanced technology of electric vehicles (EV), usually based on the power of battery with an auxiliary device. The auxiliary devices include supercapacitors (SC), flywheels, fuel cells and so on. The SC has high power density, high efficiency, fast charging and a wide range of operating advantages, especially stabilization as an auxiliary power. Because of these good characteristics, Battery/Supercapacitor HESS has gained increasing attention in energy storage community. In the Battery/Supercapacitor HESS, batteries are often used to fulfill the average power demands, whereas SCs are mainly responsible for offering transient high-power delivery. This combination can help alleviate stress on batteries during hard accelerations and regenerative braking in aggressive decelerations [1].

HESS's research involves topology design, battery modeling, supercapacitor modeling and SOC estimation, SOH prediction, DC-DC converter control strategies, power distribution strategies, and system stability analysis [2–5]. We conducted in-depth research on the power management system for electric vehicles, and then proposed a simplified cascading HESS topology configuration with the adaptive sliding mode control algorithm [6–8]. Our research has been applied to SUDA brand electric car successfully. However, excellent HESS system should also have a high-performance control strategy in addition to just having a reasonable topology.

The control for the HESS primarily focuses on the DC-DC converter. When the EV's power demand is positive, HESS will generally work in the Boost mode, with the battery (after the step-up) together with the supercapacitor to provide power for the load. When the EV needs to recover the braking energy, the HESS works (in the Buck mode) with the supercapacitor (after the step-down) to charge battery. So the control of HESS mainly involves the control of Boost and Buck converter [9]. We studied the fractional terminal sliding mode control algorithm for Buck converter, and achieved good results [10]. However, in the HESS system, the Boost mode is the main mode of operation, while the control strategy of both (HESS/Boost and Buck) modes are significantly different.

In terms of control, the fundamental control frame for a Boost type converter is a challenging work because it is a bi-linear system with a binary input in its exact description, or it can be a saturated linear system in the average model. This Boost type converter is also a minimum phase system with the output to be controlled [11–12].

Sliding mode control (SMC) is one of the effective nonlinear robust control approaches since it keeps the system dynamics controlled in the sliding mode [13]. The sliding mode control (SMC) has many advantages, such as its fast dynamic response, robustness to disturbances, guaranteed stability and simplicity in implementation [14]. There have been a lot of researches on sliding mode control for DC-DC converters. In Ref. [15], Hasan Komurcugil proposed an adaptive terminal sliding mode control strategy for Buck converter, and his sliding surface is a linear one based on linear combination of the system states(with an appropriate time-invariant coefficient). In Ref. [16–17], Wai RJ and his partners researched on in-depth study about the sliding mode control of Boost converter to improve the Boost converter control performance. In Ref. [18–20], the authors pointed out that fractional calculations apply separately to the modeling and control of DC-DC converters, but the discussion was neither specific nor in detail.

Moreover, HESS control pursuits are not only for high precision but also for the battery protection and current stabilization. Unfortunately, the resistance of the battery is not a constant value such that the external input voltage cannot be accurately calculated, and the load variation also cannot be effectively known [21–22]. In Ref. [23], the authors designed a status observer based on the estimates of the load resistor, the external input voltage, the inductor current and the output voltage.

In this paper, we focus on the high-performance control strategy for the Boost mode in the Battery/Supercapacitor HESS, and propose a novel method of adaptive fractional order sliding mode control (AFSMC). Then we utilize this method to design a novel nonlinear fractional order sliding surface function, that is a fusion of the characteristics of adaptive SMC and fractional order calculation (FOC).

The rest of the paper is organized as follows: Section 2 describes the problem statement and system modeling of the Boost mode in Battery/Supercapacitor HESS. Section 3 deals with the design of adaptive control method. Section 4 conducts the design of nonlinear controllers for the Boost mode converter based on the fractional order calculation and the adaptive sliding mode control. Section 5 shows simulation results and compares the AFSMC strategy and ASMC strategy. Finally, our conclusions are drawn in section 6.

## Problem statement and system modeling

**Fig 1:**
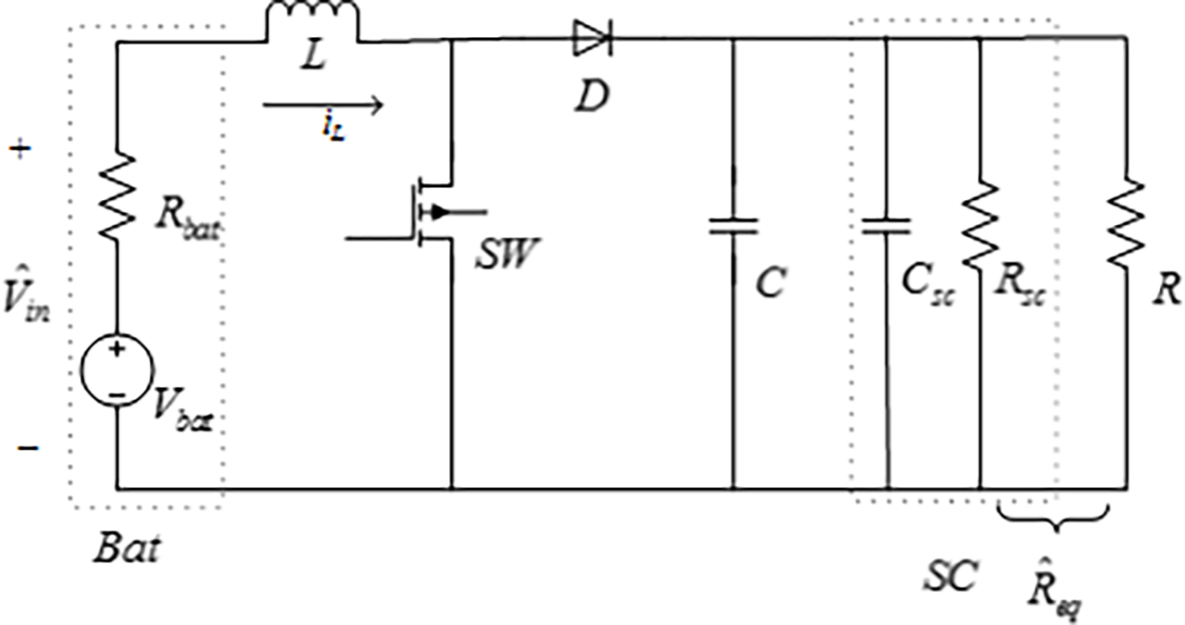
Equivalent circuit of the Battery/Supercapacitor HESS in Boost mode.

Fig 1 shows the Equivalent circuit of the Battery/Supercapacitor HESS in Boost mode, where *SW* is the main switch; *D* is the output diode; and *L*,*C*,*R* are the input inductor, output capacitor and the load resistor, respectively. The battery is equivalent to power source (*Vbat*) and small resistance (*Rbat*) as input, the supercapacitor is equivalent to capacitance (*Csc*) and large resistance (*Rsc*) as output.

By using the state-space averaging method, the average model of this equivalent circuit can be represented as

$${\dot{i}}_{L}=\frac{-(1-u)}{L}V_{o}+\frac{1}{L}V_{in}$$ (1)

$${\dot{V}}_{o}=\frac{\left(1-u\right)}{C_{eq}}i_{L}-\frac{1}{R_{eq}C_{eq}}V_{o}$$ (2)

Where *i*_*L*_ is the average inductor current, *V*_*in*_ is the input voltage which can be expressed as *V*_*in*_ = *V*_*bat*_ − *R*_*bat*_*i*_*bat*_, *V*_*bat*_ is open circuit voltage of the battery, *R*_*bat*_ is the battery resistance, *i*_*bat*_ is the battery current, *u* is the duty-ratio, *V*_*o*_ is the average output voltage.

Selection of the inductor current (*i*_*L*_) and output voltage (*V*_*o*_) as state variable of the system:

$$x_{1}=i_{L}$$ (3)

$$x_{2}=V_{o}$$ (4)

Leads to average state-space model describing the system as the following:

$${\dot{x}}_{1}=\frac{-(1-u)}{L}x_{2}+\frac{1}{L}V_{in}$$ (5)

$${\dot{x}}_{2}=\frac{\left(1-u\right)}{C_{eq}}x_{1}-\frac{1}{R_{eq}C_{eq}}x_{2}$$ (6)

## Adaptive control and adaptation rules

The control strategy of the equivalent circuit of the Battery/Supercapacitor HESS in Boost mode is similar to the one of the Boost converter. However, the changes of battery resistance and supercapacitor equivalent resistance will cause current and output voltage fluctuations. Unfortunately, the battery resistance and the supercapacitor's resistance are non-linear and dynamic. In order to reduce the magnitude of chattering and guarantee the battery safety, a constant current control for the battery should be implemented. It is necessary to design adaptive control strategy to overcome the changes in parameters caused by current and voltage fluctuations.

We define the tracking current error and the output voltage error as

$${\tilde{x}}_{1}=x_{1}-{\hat{x}}_{1}$$ (7)

$${\tilde{x}}_{2}=x_{2}-{\hat{x}}_{2}$$ (8)

Where ${\hat{x}}_{1}$ and ${\hat{x}}_{2}$ are the estimates of *x*_1_ and *x*_2_ respectively. In general ${\hat{x}}_{2}=V_{ref}$ and ${\hat{x}}_{1}=\frac{V_{ref}{}^{2}}{{\hat{V}}_{in}{\hat{R}}_{eq}}$.

Inductor current and output voltage fluctuations are mainly caused by changes in battery internal resistance and load resistance. So, we choose ${\hat{V}}_{in}$ and ${\hat{R}}_{eq}$ as estimation. Establish the observation function as

$${\dot{\hat{x}}}_{1}=\frac{-(1-u){\hat{x}}_{2}}{L}+\frac{{\hat{V}}_{in}}{L}+\alpha_{1}(x_{1}-{\hat{x}}_{1})$$ (9)

$${\dot{\hat{x}}}_{2}=\frac{(1-u){\hat{x}}_{1}}{C_{eq}}-\frac{x_{2}}{{\hat{R}}_{eq}C_{eq}}+\alpha_{2}(x_{2}-{\hat{x}}_{2})$$ (10)

Where α_1_ and α_2_ are positive integers, used to amplify the estimated value and the actual value of the error.

Define $G_{eq}=\frac{1}{R_{eq}}$, ${\tilde{G}}_{eq}=G_{eq}-{\hat{G}}_{eq}$ and ${\tilde{V}}_{in}=V_{in}-{\hat{V}}_{in}$, we can obtain

$${\dot{\tilde{x}}}_{1}=\frac{-(1-u){\tilde{x}}_{2}}{L}+\frac{{\tilde{V}}_{in}}{L}-\alpha_{1}{\tilde{x}}_{1}$$ (11)

$${\dot{\tilde{x}}}_{2}=\frac{(1-u){\tilde{x}}_{1}}{C_{eq}}-\frac{{\tilde{G}}_{eq}x_{2}}{C_{eq}}-\alpha_{2}{\tilde{x}}_{2}$$ (12)

Defining the Lyapunov function as

$$V=\frac{1}{2}L{\tilde{x}}_{1}^{2}+\frac{1}{2}(C+C_{sc}){\tilde{x}}_{2}^{2}+\frac{1}{2\beta_{1}}{\tilde{G}}_{eq}^{2}+\frac{1}{2\beta_{2}}{\tilde{V}}_{in}^{2}$$ (13)

Where β_1_ and β_2_ are the given positive constants. The time derivative of Eq (13) can be written as

$$\dot{V}=L{\tilde{x}}^{}{}_{1}{\dot{\tilde{x}}}^{}{}_{1}+(C+C_{sc}){\tilde{x}}_{2}{\dot{\tilde{x}}}_{2}+\frac{1}{\beta_{1}}{\tilde{G}}^{}{}_{eq}{\dot{\tilde{G}}}^{}{}_{eq}+\frac{1}{\beta_{2}}{\tilde{V}}^{}{}_{in}{\dot{\tilde{V}}}^{}{}_{in}$$ (14)

Substitute (11) and (12), Eq (14) can be represented as

$$\dot{V}=-L\alpha_{1}{\tilde{x}}_{1}^{2}-\alpha_{2}(C+C_{sc}){\tilde{x}}_{2}^{2}-{\tilde{G}}_{eq}(x_{2}{\tilde{x}}_{2}+\frac{{\dot{\hat{G}}}_{eq}}{\beta_{1}})+{\tilde{V}}_{in}({\tilde{x}}_{1}-\frac{{\dot{\hat{V}}}_{in}}{\beta_{2}})$$ (15)

To ensure the stability of the system, the adaptation rules are designed as ${\dot{\hat{G}}}_{eq}=-\beta_{1}x_{2}{\tilde{x}}_{2}$ and ${\dot{\hat{V}}}_{in}=\beta_{2}{\tilde{x}}_{1}$. With the adaptation rules, the Eq (15) obtain

$$\dot{V}=-L\alpha_{1}{\tilde{x}}_{1}^{2}-\alpha_{2}(C+C_{sc}){\tilde{x}}_{2}^{2}<0$$ (16)

According to the Lasalle principle, it can be know that

$${\tilde{x}}_{1}\to 0;\;{\tilde{x}}_{2}\to 0$$ (17)

In view of Eqs (9) and (10), it can conclude that ${\hat{x}}_{2}\to V_{ref}$, ${\hat{V}}_{in}\to V_{in}$, ${\hat{G}}_{eq}\to G_{eq}$ asymptotically.

## Fractional order sliding mode control

The sliding surface function is expressed as a fractional order differential equation that is obtained in the form

$$S\left(t\right)={\tilde{x}}_{1}+kD_{0}^{-\lambda }{\tilde{x}}_{1}$$ (18)

Where λ ∈ [0,1], *k* is positive constant. For the Boost mode with FSMC, the time derivative of Eq (18) can be written as

$$\dot{S}\left(t\right)=\frac{-(1-u){\tilde{x}}_{2}}{L}+\frac{{\tilde{V}}_{in}}{L}-\alpha_{1}{\tilde{x}}_{1}+kD_{0}^{-\lambda }\left(\frac{-(1-u){\tilde{x}}_{2}}{L}+\frac{{\tilde{V}}_{in}}{L}-\alpha_{1}{\tilde{x}}_{1}\right)$$ (19)

By setting $\dot{S}\left(t\right)=0$ the equivalent control is obtained, and it has the owing formula:

$$u_{eq}=1-\frac{{\tilde{V}}_{in}-\alpha_{1}L{\tilde{x}}_{1}}{{\tilde{x}}_{2}}$$ (20)

Then, the global control is given by:

$$u=1-\frac{{\tilde{V}}_{in}-\alpha_{1}L{\tilde{x}}_{1}}{{\tilde{x}}_{2}}+K\cdot D_{0}^{-\lambda }\left(\mathrm{sgn}\left(S\right)\right)$$ (21)

Substituting of (21) in (19) results:

$$\dot{S}\left(t\right)=-K\cdot D_{0}^{-\lambda }\left(\mathrm{sgn}\left(S\left(t\right)\right)\right)-K\cdot D_{0}^{-\lambda }\left(\mathrm{sgn}\left(S\left(0\right)\right)\right)$$ (22)

For initial condition *x*_1_ = 0, the sliding surface *S*(0) = 0, then Eq (22) can be rewritten as the following:

$$\dot{S}\left(t\right)=-K\cdot D_{0}^{-\lambda }\left(\mathrm{sgn}\left(S\left(t\right)\right)\right)$$ (23)

By selecting the Lyapunov function as

$$V=S^{2}$$ (24)

The time derivative of Eq (24) can be written as

$$\dot{V}=2S\dot{S}$$ (25)

Using Eq (23), then Eq (25) can be obtained as

$$\dot{V}=2S\left(t\right)\dot{S}\left(t\right)=-2K\cdot S\cdot D_{0}^{-\lambda }\left(\mathrm{sgn}\left(S\right)\right)\leq 0$$ (26)

As can be seen from Eq (26), the proposed sliding surface can satisfy the stability condition.

Furthermore, through the fractional version of Lyapunov by direct method, the same relationship can also be proven [24]. It follows from the Ref. [25], if 0 is the equilibrium point of system (21) and *x*(0) = *x*_0_, then the fractional order derivative of Eq (24), can be written as

$$\begin{array}{l} D^{1-\lambda }V=D^{-\lambda }\dot{V}\leq -KD^{-\lambda }\left\|x_{1}\right\|\\ =-Kl^{-1}D^{-\lambda }\left\|S\right\|\leq -Kl^{-1}\left\|D^{-\lambda }S\right\|\\ =-Kl^{-1}\left\|x_{1}\right\| \end{array}$$ (27)

Where *K* is positive constant, *l* is Lipschitz constant and *l* > 0. So, we can find *V* > 0 and *D*^1−λ^*V* < 0. In other words, the controlled system satisfies the reaching condition.

## Simulation and discussion

In order to show the performance of the AFSMC, the equivalent circuit of Boost mode system was subsequently tested by simulations. Simulations are carried out using MATLAB/Simulink. The parameters of equivalent circuit are given in Table 1.

**Table 1:** Specifications of equivalent circuit of the Battery/Supercapacitor HESS in Boost mode.

Descriptions	Parameters	Nominal values
Battery	*Vbat*,*C*	25V, 55Ah
Supercapacitor	*Vsc*,*Csc*	50 V, 15.7F
Desired output voltage	*Vref*	50V
Inductance	*L*	260 uH
Capacitance	*C*	100 uF
Load resistance	R	5Ω

When the supercapacitor's open circuit voltage and SOC are low, we approximate that *V*_*bat*_ = 25 V, *V*_*sc*_ = 0 V, *V*_*ref*_ = 50 V, in this simulation study. Further, we investigated the dynamic response of output voltage with different fractional orders (*λ*) on the basis of the adaptive fractional order sliding mode control strategy. The control gains of the AFSMC system are given as follows: α_1_ = 6*G*_*eq*_ / *C*, α_2_ = 2*G*_*eq*_ / *C*, β_1_ = 2, β_2_ = 100, *k* = 300.

**Fig 2:**
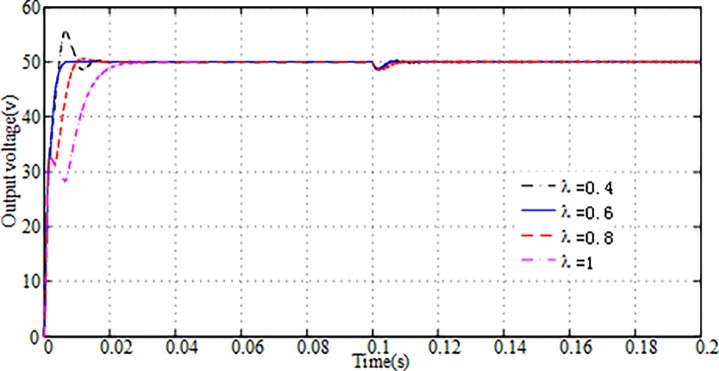
Simulated output voltage responses due to the different λ by AFSMC.

Fig 2 shows the simulated start-up and transient responses of the output voltage obtained by AFSMC strategies with different λ values. It is interesting to note that the output voltage responses become faster with decreasing the value of λ, but when λ = 0.4, the overshoot of the system appears and exceeds 10%. In order to ensure the safety of SC and obtain high performance control strategy, we should try to avoid the voltage overshoot and chattering. And at *𝑡* = 0.1s, the load resistance is changed from 5Ω to 1 Ω. Therefore, the output current will be increased for this *t*-value, and the output voltage has a short step-down. It can be seen that the actual output voltage returns faster to desired voltage with decreasing the value of λ, too. So λ = 0.6 is our choice for ideal parameter value.

**Fig 3:**
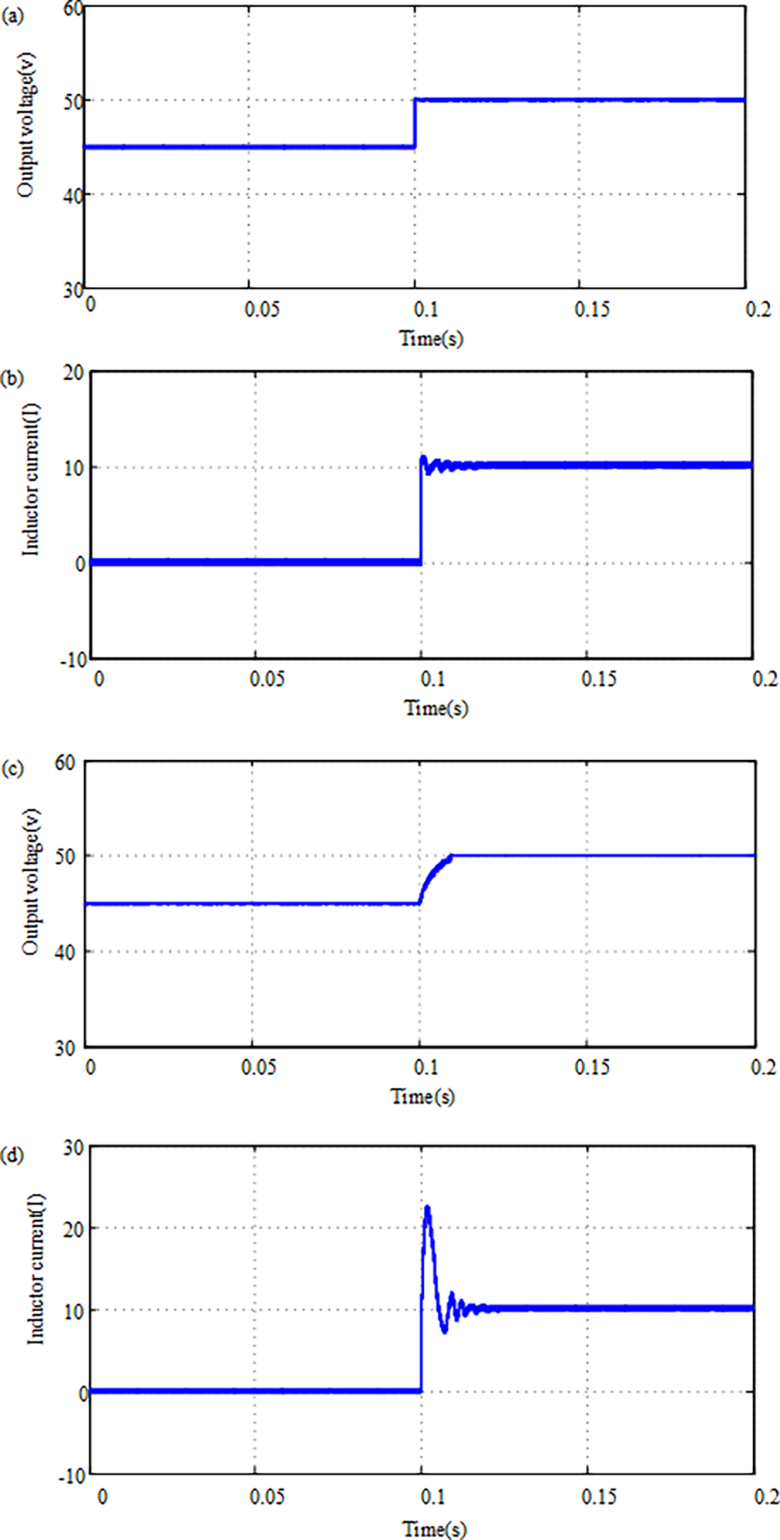
**Simulation results of the FASMC and ASMC strategy for the Battery/supercapacitor HESS:** (a) The turn-on voltage of the FASMC; (b) The inductor current of the FASMC; (c)The turn-on voltage of the ASMC; (d) The inductor current of the ASMC.

When the SOC of supercapacitor is high, we approximate that *V*_*bat*_ = 25 V, *V*_*sc*_ = 45 V, *V*_*ref*_ = 50 V simulation study. At *t* = 0.1s, we start the converter and make the output 45V boost to 50V. We investigated and compared AFSMC (λ = 0.6) and ASMC (λ = 1), respectively. The output voltage response and the inductor current at conditions AFSMC and ASMC are shown in Fig 3. From Fig 3(A)–3(D), we can see that both control strategies have in-rush current at start-up, which is caused by the MOS and capacitor. However, the current is significantly more stable in AFSMC strategy. In addition, it can be known that the AFSMC strategy can reduce the adjusting time of the turn-on voltage, as compared with the ASMC strategy. It can improve up to 80% of the transient time during startup of the Boost mode converter.

## Conclusions

The application of a AFSMC system to achieve a steady inductor current and control output voltage of the Boost mode in Battery/supercapacitor HESS has been successfully demonstrated. Fristly, the description of the equivalent circuit of the Battery/supercapacitor HESS in Boost mode and system modeling was introduced. Then, the design procedure and the stability analyses of the proposed AFSMC scheme were described in detail. Moreover, numerical simulations in different operational conditions were carried out, and the dynamic response of output voltage with different fractional orders were investigated. According to the simulation results, when the fractional order (*λ*) equals to 0.6, the performance of dynamic responses is better than the other values of λ. Compared with the integer order ASMC strategy, the fractional order ASMC method decreases 10% of the transient time to deal with the load variation. It also reduces 80% of the transient time during the startup of the Boost mode converter. Therefore, the AFSMC strategy not only provides a constant current, but also allows the HESS system to reach a steady state quickly. So, this application of the AFSMC strategy to the HESS of an electric vehicle will improve the overall performance.
